# Intra-study and inter-technique validation of cardiovascular magnetic resonance imaging derived left atrial ejection fraction as a prognostic biomarker in heart failure with preserved ejection fraction

**DOI:** 10.1007/s10554-020-01785-w

**Published:** 2020-02-06

**Authors:** Prathap Kanagala, Jayanth R. Arnold, Anvesha Singh, Jamal N. Khan, Gaurav S. Gulsin, Pankaj Gupta, Iain B. Squire, Leong L. Ng, Gerry P. McCann

**Affiliations:** 1 Department of Cardiovascular Sciences, University of Leicester, National Institute for Health Research (NIHR) Leicester Biomedical Research Centre, Leicester, UK; 2 Aintree University Hospital, Liverpool, UK; 3 Department of Cardiovascular Sciences, Glenfield Hospital, Groby Road, Leicester, LE3 9QP UK

**Keywords:** Heart failure with preserved ejection fraction, Prognosis, Left atrial ejection fraction, Biplane, Short-axis

## Abstract

The aim of this study was to assess the agreements of both biplane and short-axis Simpson’s (SAX) methods for left atrial ejection fraction (LAEF) calculation utilising cardiovascular magnetic resonance imaging (CMR) in heart failure with preserved ejection fraction (HFpEF) and evaluate their relation to clinical outcomes. One hundred and thirty six subjects (HFpEF n = 97, controls n = 39) underwent CMR, six-minute walk tests and blood sampling in our prospective, observational, single-centre study. Overall, LAEF (%) was lower in HFpEF patients compared to controls (SAX 34 ± 13 vs 47 ± 8, biplane 34 ± 16 vs 51 ± 11; p < 0.0001 for both). Atrial fibrillation (AF) was present in 24% of HFpEF and was associated with higher LA volumes and lower LAEF compared to sinus rhythm (p < 0.0001) with both methods. Biplane LAEF correlated strongly with SAX measurements (overall Pearson’s r = 0.851, sinus rhythm r = 0.651, AF r = 0.882; p < 0.0001). Biplane LAEF did not differ significantly compared to SAX LAEF (overall 34 ± 16 vs 34 ± 13%; p = 0.307) except in AF subjects in whom biplane LAEF was lower (mean difference 2 ± 4%, p = 0.013). There were 44 composite events (25 deaths, 19 HF hospitalizations) in HFpEF during median follow-up of 1429 days. LAEF below the median was associated with increased risk of composite endpoints (Log-Rank biplane p < 0.0001; SAX p = 0.009). In multivariable Cox proportional hazards regression analysis, both biplane LAEF (hazard ratio [HR] 0.604; 95% confidence interval [CI] (0.406–0.900); p = 0.013) and SAX LAEF (HR 0.636; CI 0.441–0.918; p = 0.016) remained independent predictors along with indexed extracellular volume. CMR LAEF, derived from either the short-axis or biplane method is lower in HFpEF compared to healthy controls and remains a strong marker of prognosis.

## Introduction

In our recently published article [1], we reported that cardiovascular magnetic resonance (CMR) derived left atrial ejection fraction (LAEF) measured from the biplane method was lower in a well characterized cohort of heart failure with preserved ejection fraction (HFpEF) compared to healthy controls and was also independently associated with adverse outcomes. Furthermore, CMR biplane LAEF had excellent reproducibility. However, volumetric assessment of left atrial volumes (and hence function) derived from the short-axis (Simpson’s short-axis [SAX]) method is widely recognised as the imaging gold standard [2, 3]. In a recent study of HFpEF patients, the biplane method was shown to have good correlation and agreements for LA volumetric analysis compared to the SAX method [4]. However, no prior CMR studies in HFpEF have compared LAEF between both methods. Furthermore, the prognostic role of SAX LAEF in HFpEF has not been reported. We aimed to assess the agreements of both methods for LAEF (and LA volumes) utilising CMR in HFpEF. We also evaluated whether SAX LAEF is also related to clinical outcomes, in order to validate and strengthen our previous findings implicating CMR LAEF as a prognostic biomarker in HFpEF.

## Methods

From our original study cohorts [1] of HFpEF (n = 140) and healthy controls (n = 48), paired data for image analysis of both CMR biplane and SAX LAEF derivation was available in 136 subjects (HFpEF n = 97, controls n = 39). HFpEF was defined as clinical or radiographic evidence of heart failure and left ventricular ejection fraction > 50%. Exclusion criteria for HFpEF included: myocardial infarction in the preceding 6 months, suspected or confirmed cardiomyopathy or constrictive pericarditis, non-cardiovascular life expectancy < 6 months, severe chronic obstructive pulmonary disease (or forced expiratory volume [FEV_1_] < 30% predicted or forced vital capacity [FVC] < 50% predicted), severe native valve disease and significant renal impairment (estimated glomerular filtration rate [eGFR] < 30 ml/min. The control population were age- and sex-matched compared to HFpEF and asymptomatic. As previously reported, hypertensive controls were also included since hypertension is highly prevalent in this age group of patients. Furthermore, hypertension is intimately linked with HFpEF development and is also reportedly associated with LA dysfunction [5] and we wanted to account for this potential confounder.

**Fig. 1:**
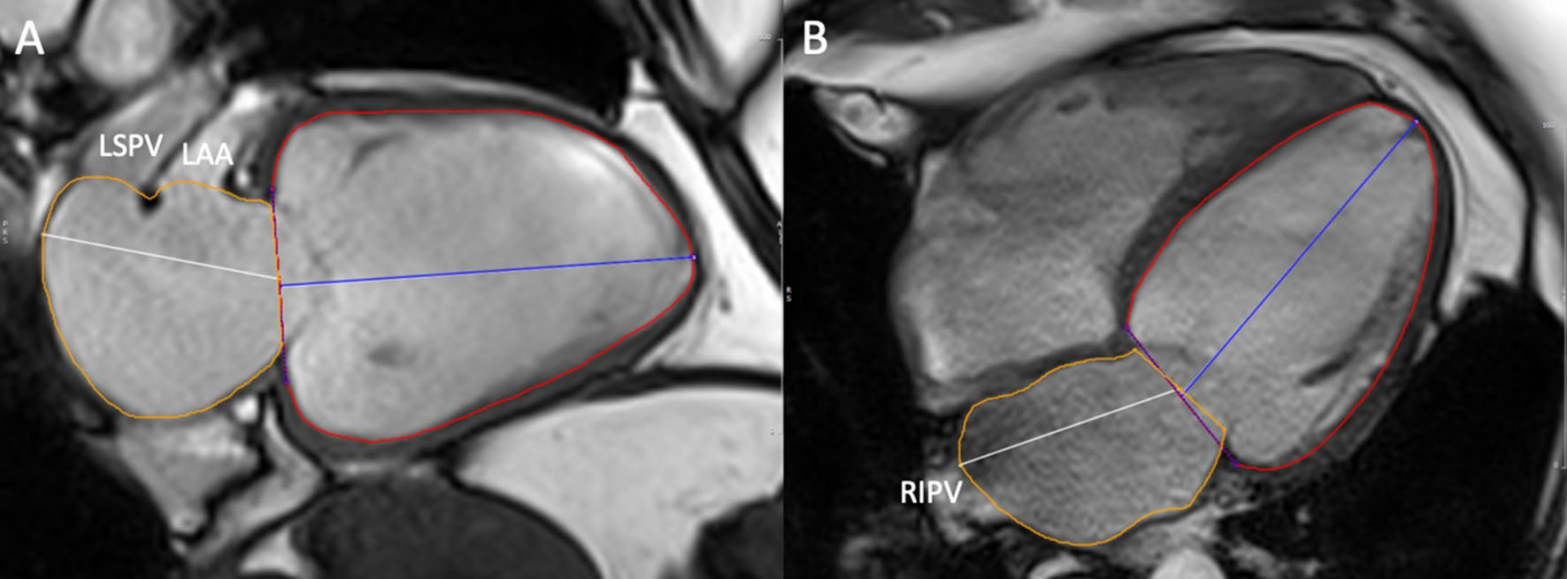
Biplane method. Cine 2- (**a**) and 4-chamber (**b**) images illustrating contoured (yellow) left atrial areas for volume (and ejection fraction) derivation excluding the left atrial appendage and pulmonary veins; *LAA* left atrial appendage; *LSPV* left superior pulmonary vein; *RIPV* right inferior pulmonary vein

**Fig. 2:**
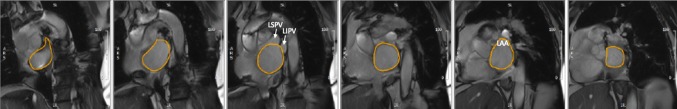
Simpson’s short-axis method. Short-axis cine stack of images illustrating contoured (yellow) left atrial areas for volume (and ejection fraction) derivation inclusive of the left atrial appendage but excluding the pulmonary veins. *LAA* left atrial appendage, *LSPV* left superior pulmonary vein; *LIPV* left inferior pulmonary vein

Study recruitment, blood sampling, six-minute walk testing, imaging protocols and analytical methods for CMR have been detailed previously. For the SAX method (see Fig. 1), a contiguous stack of short-axis steady-state-free-precession images were acquired with retrospective ECG gating (or prospective gating in subjects with AF). Both end-diastolic and end-systolic frames were contoured to derive maximal left atrial volume [LAV max] and minimal left atrial volume (LAV min) and LAEF was calculated. Unlike the biplane method (see Fig. 2), SAX volumes were inclusive of the LA appendage but both techniques excluded pulmonary veins. The primary endpoint remained the composite of all-cause mortality or first HF hospitalization. Parameters with univariable association with the composite endpoint at p < 0.1 was entered into Cox proportional hazards regression analysis. In cases of collinearity, variables with historically stronger prognostic importance from published literature were chosen for inclusion in Cox regression analysis and underwent stepwise elimination. Four separate clinically relevant Cox regression models were generated including a final model incorporating the strongest predictors. Cox regression models were limited to no more than 4 parameters (plus LAEF), allowing for approximately one parameter per 10 composite events. To allow hazard ratios (HR) to be compared according to one standard deviation increase, continuous predictor variables were Z-standardized. Event rates were calculated from Kaplan–Meier analysis. The Log-Rank test was used to detect differences in survival curves. In order to further assess the strength of both biplane and SAX LAEF in predicting outcomes, receiver operator characteristics (ROC) analyses were also performed.

Inter-technique comparison of both LAEF derivation methods included paired t-testing, regression and the Bland–Altman method. Ten randomly selected subjects were chosen to undergo intra-observer evaluation for both techniques, a minimum of 4 weeks apart. The two-way mixed-effect intraclass correlation co-efficient (ICC), coefficient of variation (COV) and Bland–Altman analysis were used for test intra-observer agreements.

## Results

Baseline clinical and imaging characteristics are shown in Tables 1 and 2. Both HFpEF and controls remained evenly matched for age (73 ± 8) and gender. HFpEF was associated with a high burden of co-morbidities including hypertension, diabetes, anaemia and renal dysfunction. Atrial fibrillation (AF) was noted in 24% of HFpEF. Compared to controls, HFpEF was characterised by lower exercise capacity, worse diastolic function (LA volumes, left ventricular mass, B-type natriuretic peptide [BNP] and N-terminal pro-atrial natriuretic peptide [NT-proANP]), more prevalent focal (late gadolinium enhancement imaging) and diffuse fibrosis (extracellular volume and indexed extracellular volume [iECV]); p < 0.05 for all. Feasibility of image analysis for LA measures by both techniques was 100% in all subjects. With both methods, HFpEF patients had lower LAEF (biplane 34 ± 16; SAX 34 ± 13) compared to controls: overall (biplane 51 ± 11, SAX 47 ± 8), with hypertension (biplane 48 ± 12, SAX 45 ± 9) and without hypertension (biplane 52 ± 9, SAX 49 ± 7); p < 0.0001 for all. No significant differences in LAEF were noted between hypertensive and non-hypertensive controls (biplane p = 0.222, SAX p = 0.119). Irrespective of methodology and cardiac rhythm, HFpEF was characterised by significantly higher LA volumes compared to controls (p < 0.0001). HFpEF patients in AF had higher LA volumes and lower LAEF compared to sinus rhythm (p < 0.0001).

**Table 1:** Baseline clinical characteristics

	HFpEF n = 97	Controls n = 39	P value
Demographics			
Age (years)	72 ± 10	73 ± 5	0.486
Male (%)	52 (54)	18 (46)	0.682
Clinical			
Heart rate (beats per minute)	71 ± 14	68 ± 10	0.133
Systolic blood pressure (mmHg)	145 ± 23	151 ± 24	0.155
Diastolic blood pressure (mmHg)	74 ± 12	79 ± 10	0.012
Body mass index (kg/m^2^)	34 ± 8	25 ± 3	< 0.0001
Atrial fibrillation (%)	23 (24)	0 (0)	< 0.0001
Prior HF hospitalization (%)	62 (64)	0 (0)	< 0.0001
Diabetes (%)	55 (57)	0 (0)	< 0.0001
Hypertension (%)	89 (92)	19 (49)	< 0.0001
Angina (%)	15 (16)	0 (0)	0.004
Known myocardial infarction (%)	12 (12)	0 (0)	0.011
Asthma or COPD (%)	14 (14)	3 (8)	0.149
TIA or CVA (%)	12 (12)	0 (0)	0.011
Functional status			
NYHA III/IV (%)	23 (24)	NA	–
6MWT distance (m)	190 (130–275)	380 (340–440)	< 0.0001
MLHF score	49 (24–65)	NA	–
Medications			
Betablocker (%)	67 (69)	2 (5)	< 0.0001
ACEi or ARB (%)	86 (89)	9 (23)	< 0.0001
Aldosterone antagonist (%)	30 (31)	0 (0)	< 0.0001
Loop diuretic (%)	77 (79)	0 (0)	< 0.0001
Laboratory indices			
Urea (mmol/L)	9 ± 4	6 ± 1	< 0.0001
Creatinine (umol/L)	92 (74–118)	67 (56–85)	< 0.0001
Haemoglobin (g/L)	131 ± 23	140 ± 15	0.003
BNP (ng/L)	117 (51–244)	33 (24–44)	< 0.0001
NTpro-ANP (pg/ml)	6321 (3874)	4246 (3402–4532)	< 0.0001

Values are mean ± SD, n (%) or median, interquartile range. The p values are for the t test or chi-square test

*BNP* B-type natriuretic peptide, *COPD* chronic obstructive pulmonary disease, *HF* heart failure, *HFpEF* heart failure with preserved ejection fraction, *NA* not applicable, *NTpro-ANP* N-terminal pro-atrial natriuretic peptide, *NYHA* New York Heart Association class, *6MWT* six minute walk test

**Table 2:** Baseline imaging characteristics

	HFpEF n = 97	Controls n = 39	p value
CMR LV parameters			
LVEF (%)	56 ± 5	58 ± 5	0.058
LVEDVI (ml/m^2^)	79 ± 18	83 ± 14	0.237
LVESVI (ml/m^2^)	35 ± 10	35 ± 8	0.950
LV mass indexed (g/m^2^)	52 ± 15	46 ± 10	0.003
LV mass/LVEDV	0.68 ± 0.16	0.57 ± 0.09	< 0.0001
LV tissue characterisation			
Presence of MI (%)	13 (13)	0 (0)	0.007
MI size (% of LV mass)	3.0 (0.9–4.9)	NA	–
Presence of non-MI focal fibrosis (%)	38 (39)	5 (13)	0.007
Non-MI fibrosis size (% of LV mass)	2.8 (1.2–6.6)	2.4 (0.6–3.6)	< 0.0001
^a^Native myocardial T1 (ms)	1230 ± 78	1191 ± 98	0.038
^a^Post-contrast myocardial T1 (ms)	457 ± 61	489 ± 93	0.007
^a^ECV (%)	28 ± 5	26 ± 3	0.008
^a^iECV (ml/m^2^)	14 ± 4	11 ± 3	0.001
CMR LA parameters			
Overall—all subjects including atrial fibrillation			
SAX LAV max (ml)	120 ± 50	88 ± 21	< 0.0001
SAX LAV min (ml)	84 ± 49	47 ± 14	< 0.0001
SAX LAEF (%)	34 ± 13	47 ± 8	< 0.0001
Biplane LAV max (ml)	102 ± 44	62 ± 22	< 0.0001
Biplane LAV min (ml)	71 ± 44	31 ± 14	< 0.0001
Biplane LAEF (%)	34 ± 16	51 ± 11	< 0.0001
Sinus			
SAX LAV max (ml)	103 ± 33	88 ± 21	0.004
SAX LAV min (ml)	64 ± 26	47 ± 14	< 0.0001
SAX LAEF (%)	39 ± 10	47 ± 8	< 0.0001
Biplane LAV max (ml)	88 ± 32	62 ± 22	< 0.0001
Biplane LAV min (ml)	53 ± 25	31 ± 14	< 0.0001
Biplane LAEF (%)	41 ± 12	51 ± 11	< 0.0001
Atrial fibrillation			
SAX LAV max (ml)	175 ± 56	NA	–
SAX LAV min (ml)	148 ± 51	NA	–
SAX LAEF (%)	16 ± 8	NA	–
Biplane LAV max (ml)	147 ± 45	NA	–
Biplane LAV min (ml)	129 ± 43	NA	–
Biplane LAEF (%)	14 ± 9	NA	–

*ECV* extracellular volume, *iECV* indexed to body surface area; extracellular volume, *LA* left atrium, *LAEF* left atrial ejection fraction, *LAV max* maximal left atrial volume, *LAV min* minimal left atrial volume, *LV* left ventricle, *LVEDVI* left ventricular end-diastolic volume indexed to body surface area, *LVEF* left ventricular ejection fraction, *LVESVI* left ventricular end-systolic volume indexed to body surface area, *MI* myocardial infarction

^a^Available in n = 72 HFpEF, n = 35 controls

Biplane LA measures correlated strongly with SAX parameters overall (LAV max Pearson’s r = 0.910, LAV min r = 0.884, LAEF r = 0.851), in sinus rhythm (LAV max 0.926, LAV min r = 0.920, LAEF r = 0.651) and moderate to good in AF (LAV max r = 0.776, LAV min r = 0.788, LAEF r = 0.882); p < 0.0001 for all. LA volumes calculated from the biplane method (excluding the LA appendage) were significantly lower compared to the SAX method overall and irrespective of whether subjects were in sinus rhythm or AF (Table 2). While overall agreements for LA volumes were good (Table 3), the limits of agreement were wider in AF. Biplane LAEF did not differ significantly compared to SAX LAEF except in AF subjects in whom biplane LAEF was comparably lower (mean difference 2 ± 4%, p = 0.013). Intra-observer agreements for both methods were excellent, albeit biplane measures fared slightly worse (Table 4).

**Table 3:** Inter-technique agreements for left atrial volumes and ejection fraction between CMR short-axis and biplane methods

Parameter	CMR SAX mean ± SD	CMR biplane Mean ± SD	Mean dfference ± SD	95% Limits of agreement	P value
All patients (n = 97)					
LAV max (ml)	120 ± 50	102 ± 44	18 ± 21	− 22 to 60	< 0.0001
LAV min (ml)	84 ± 49	71 ± 44	13 ± 18	− 22 to 48	< 0.0001
LAEF (%)	34 ± 13	34 ± 16	− 1 ± 8	− 17 to 15	0.307
Sinus rhythm (n = 74)					
LAV max (ml)	103 ± 33	88 ± 32	15 ± 13	− 9 to 41	< 0.0001
LAV min (ml)	64 ± 26	53 ± 25	11 ± 10	− 9 to 31	< 0.0001
LAEF (%)	39 ± 10	41 ± 12	− 2 ± 9	− 20 to 16	0.083
Atrial fibrillation (n = 23)					
LAV max (ml)	176 ± 56	147 ± 45	27 ± 35	− 42 to 97	0.001
LAV min (ml)	148 ± 51	129 ± 43	19 ± 32	− 43 to 81	0.008
LAEF (%)	16 ± 8	14 ± 9	2 ± 4	− 6 to 10	0.013

Abbreviations are as for Table 2

**Table 4:** Intra-observer assessments for left atrial volumes and ejection fraction

Parameter	Observer 1 Mean ± SD	Observer 2 Mean ± SD	Mean difference ± SD	ICC	Variability (1—ICC)	Co-efficient of variation	95% Limits of agreement
Intra-observer							
SAX							
LAV max (ml)	123 ± 40	122 ± 39	1 ± 3	0.99	0.01	3	− 7 to 5
LAV min (ml)	85 ± 44	84 ± 43	1 ± 2	0.99	0.01	2	− 4 to 3
LAEF (%)	34 ± 14	34 ± 14	0 ± 2	0.99	0.01	6	− 4 to 4
Biplane							
LAV max (ml)	99 ± 48	101 ± 49	2 ± 5	0.99	0.01	4.8	− 7 to 12
LAV min (ml)	70 ± 45	71 ± 44	1 ± 4	0.99	0.01	5.4	− 7 to 8
LAEF (%)	33 ± 13	33 ± 13	0.1 ± 3	0.98	0.02	9.4	− 6 to 6

Abbreviations are as for Table 2

*ICC* intra-class correlation coefficient

**Fig. 3:**
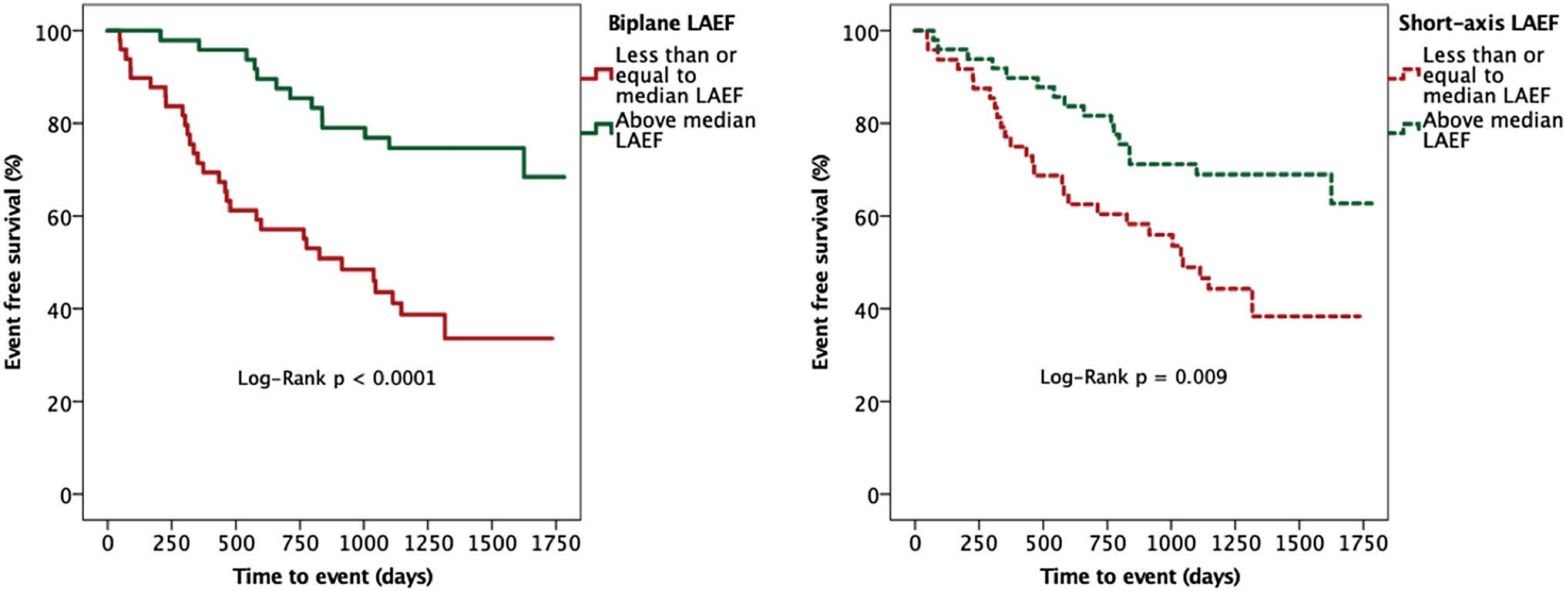
Survival analysis stratified according to median left atrial ejection fraction. Kaplan–Meier analysis stratified according to median left atrial ejection fraction for the composite endpoint of death and/or hospitalization with heart failure using the biplane method (left panel) and the short-axis method (right panel)

Forty four composite events (45%, 25 deaths, 19 HF hospitalizations) were observed in the HFpEF group during median follow-up of 1429 days (1157–1657). Sixteen parameters were associated with outcomes during Cox regression analysis, including both biplane and SAX LAEF (Table 5). Urea and extracellular volume (ECV) were excluded from regression analysis due to collinearity. On Cox regression analysis (Table 6), both biplane and SAX LAEF remained significantly associated with outcome in 3 separate models incorporating clinical factors, biochemical markers and imaging parameters. In a final model comprising the strongest predictors overall, biplane LAEF (HR 0.604; 95% confidence interval [CI] (0.406–0.900); p = 0.013) and SAX LAEF (HR 0.636; CI 0.441–0.918; p = 0.016) remained independent predictors along with indexed extracellular volume (iECV). Irrespective of methodology, a lower LAEF group (below median) was associated with increased risk of the composite endpoint (biplane Log-Rank p < 0.0001; SAX Log-Rank p = 0.009); see Fig. 3. The area under curve for predicting outcomes was higher for SAX LAEF (0.709, p < 0.0001), albeit not significantly different compared to biplane LAEF (0.690, p = 0.001).

**Table 5:** Unadjusted predictors for the composite endpoint of death and/or hospitalization with heart failure

Unadjusted predictors of outcome		
	Hazard ratio (95%CI)	P value
Clinical		
Age (years)	1.630 (1.203–2.210)	0.002
Average diastolic BP (mmHg)	0.562 (0.388–0.814)	0.002
Prior HF hospitalization	2.236 (1.104–4.529)	0.025
6MWT distance (m)	0.545 (0.339–0.876)	0.012
Clinical blood samples		
^a^Urea (mmol/L)	1.284 (1.002–1.644)	0.048
Log creatinine (umol/L)	1.352 (1.024–1.784)	0.033
Haemoglobin (g/L)	0.766 (0.576–1.019)	0.067
Log BNP (ng/L)	1.542 (1.086–2.189)	0.016
NTpro-ANP (pg/ml)	1.551 (1.113–2.160)	0.009
Imaging (CMR)		
LV mass index (g/m^2^)	1.432 (1.020–2.010)	0.038
LAVI max (ml/m^2^)	1.595 (1.202–2.115)	0.001
LGE MI (%)	1.687 (0.784–3.632)	0.181
^a^ECV (%)	1.822 (1.173–2.831)	0.008
iECV (ml/m^2^)	1.558 (1.097–2.213)	0.013
Biplane LAEF (%)	0.575 (0.419–0.788)	0.001
SAX LAEF (%)	0.596 (0.447–0.794)	0.0001

Abbreviations are as for Tables 1 and 2; Hazard ratios are based upon one standard deviation increase in the predictor variable for continuous variables which are Z-standardized

*CI* confidence interval; *LAVI max* left atrial maximal volume indexed to body surface area

^a^Parameters not entered into multivariable analysis

**Table 6:** Multiple Cox regression models inclusive of biplane and SAX LAEF for the composite endpoint of death and/or hospitalization with heart failure

Cox proportional hazard regression predictors of outcome					
Including biplane LAEF			Including SAX LAEF		
	Hazard ratio (95%CI)	P value		Hazard ratio (95%CI)	P value
Clinical					
Age	1.126 (0.771–1.645)	0.538	Age	1.049 (0.708–1.555)	0.811
Average diastolic BP	0.606 (0.407–0.904)	0.014	Average diastolic BP	0.580 (0.390–0.863)	0.007
Prior HF hospitalization	1.693 (0.810–3.540)	0.162	Prior HF hospitalization	1.588 (0.754–3.347)	0.224
6MWT distance	0.596 (0.372–0.956)	0.032	6MWT distance	0.613 (0.387–0.972)	0.038
** + Biplane LAEF**	0.535 (0.385–0.744)	< 0.0001	** + SAX LAEF**	0.532 (0.391–0.724)	< 0.0001
Clinical blood samples					
Log creatinine (umol/L)	1.035 (0.731–1.466)	0.0847	Log creatinine (umol/L)	1.033 (0.734–1.454)	0.853
Haemoglobin (g/L)	0.805 (0.584–1.109)	0.155	Haemoglobin (g/L)	0.677 (0.496–0.923)	0.114
Log BNP (ng/L)	1.126 (0.712–1.782)	0.611	Log BNP (ng/L)	1.128 (0.712–1.787)	0.607
Log NTpro-ANP	1.373 (0.990–1.906)	0.058	NTpro-ANP	1.244 (0.890–1.739)	0.202
** + Biplane LAEF**	0.649 (0.456–0.924)	0.016	** + SAX LAEF**	0.552 (0.410–0.741)	< 0.0001
Imaging					
LV mass index	0.582 (0.217–1.560)	0.282	LV mass index	0.529 (0.205–1.366)	0.188
LAVI max	0.961 (0.599–1.541)	0.869	LAVI max	0.997 (0.630–1.577)	0.988
iECV	1.558 (1.097–2.213)	0.013	iECV	1.564 (1.106–2.211)	0.011
** + Biplane LAEF**	0.575 (0.419–0.788)	0.001	** + SAX LAEF**	0.668 (0.472–0.944)	0.022
Strongest markers combined					
Average diastolic BP	0.723 (0.461–1.134)	0.158	Average diastolic BP	0.725 (0.461–1.139)	0.163
6MWT distance	0.611 (0.354–1.053)	0.076	6MWT distance	0.592 (0.346–1.011)	0.055
iECV	1.491 (1.038–2.143)	0.031	iECV	1.584 (1.110–2.260)	0.011
** + Biplane LAEF**	0.604 (0.406–0.900)	0.013	** + SAX LAEF**	0.636 (0.441–0.918)	0.016

## Discussion

Our study is the first to compare the agreements of both CMR biplane and SAX LAEF in HFpEF and assess their prognostic capabilities in the same setting. Our results confirm LAEF as an independent marker of prognosis in HFpEF, irrespective of CMR technique and validate our earlier findings of biplane LAEF as a strong prognostic biomarker [1]. The ability of CMR LAEF to be measured via 2 separate methods with a high degree of precision and reproducibility in addition to its prognostic capabilities are important strengths for consideration when being proposed as an imaging biomarker [6]. While CMR SAX LAEF is considered the more accurate of both measures [2, 3], the CMR biplane method represents a potentially useful alternative for both research and clinical settings in HFpEF and has been recently proposed as the preferred method of LA volumetric analysis in an expert consensus document by the European Association of Cardiovascular Imaging [7]. Biplane LAEF derivation does not necessitate additional, multiple short axis image acquisitions which can prolong scan times in predominantly elderly subjects typical of HFpEF, who may struggle with multiple breath-holds. Furthermore, image analysis of the biplane method is also significantly shorter. On the other hand, SAX LAEF is an alternative if long axis images are degraded by artefact. Our findings are also important since LAEF represents a potential therapeutic target in HFpEF [8] where there are currently no effective therapies [9] and may also act as a trial endpoint [10].

While LA volumes were underestimated by the biplane method compared to SAX evaluation, LAEF did not differ significantly except in AF. Since LAEF is a fraction derived from volumetric analysis, systematic bias of similar magnitudes in the same direction i.e. underestimation of both LAV max and LAV min likely explains this lack of LAEF difference between both the techniques. The difference in LA volumes between both methods however is unsurprising given that the LA appendage is typically excluded from the biplane method but included in the SAX method [2, 3]. These volumetric differences are likely further exaggerated in HFpEF where LA dilation is typical [2, 4] and associated with a higher prevalence of AF. Furthermore, in AF we employed prospective gating which can lower the margins for erroneous measurements. As addressed in our recent publication, we also recognise that having some subjects with hypertension in our control group is a limitation given that these subjects were not totally free from cardiovascular disease. However, LAEF was again lower in HFpEF with either methodology, irrespective of controls’ hypertensive status, reinforcing altered LA contractile function in the pathophysiology of HFpEF.

## Conclusions

CMR LAEF calculated from either the short-axis or biplane method has excellent reproducibility and remains a strong marker of prognosis in HFpEF.
